# Staff ratings of occupational engagement among people with severe mental illness – psychometric properties of a screening tool in the day center context

**DOI:** 10.1186/s12913-017-2283-3

**Published:** 2017-05-08

**Authors:** Mona Eklund, Ulrika Bejerholm

**Affiliations:** Department of Health Sciences/ Mental Health, Activity and Participation, Lund University, Box 157, SE 221 00 Lund, Sweden

**Keywords:** Factor analysis, Construct validity, Homogeneity, Community-based psychiatry

## Abstract

**Background:**

Staff who plan and organize day center activities may need to observe the attendees’ performance and progression. This led us to develop a tool for that purpose, termed General Occupational Engagement in people with Severe mental illness (GOES). The aim was to investigate its psychometric properties in terms of factor structure, internal consistency, corrected item-total correlations (CITC), convergent and discriminant validity, and test-retest stability.

**Methods:**

Ninety-three day center attendees were assessed by the GOES and instruments addressing constructs hypothesized to be either similar to (activity level, motivation for day center attendance, perceptions of the worker role, hours spent in the day center) or divergent from the GOES (attendees’ ratings of engagement in specified occupations, self-rated health, psychosocial functioning, psychiatric symptoms). A second sample of 41 attendees were included for the test-retest analysis. Exploratory factor analysis, Cronbach’s alpha analysis, Pearson correlations and paired-samples *t*-tests were performed.

**Results:**

Exploratory factor analysis indicated one factor, which was in line with the intentions of the scale. The alpha value was 0.85 and all CITC were above 0.30. The tests for convergent validity resulted in correlations ranging between 0.23 and 0.47, most of which were moderately strong and mainly confirmed the hypotheses. Discriminant validity was clearly indicated, since all correlations with the selected constructs were <0.20. GOES also showed preliminary test-retest stability (*r* = 0.32).

**Conclusions:**

The GOES is ready for use in rehabilitation services and research where productive and other types of activities are of interest. It may serve as an important supplement to attendees’ self-reported occupational engagement.

## Background

People with severe mental illness often lack work or other productive and meaningful activities [1–3]. Most people in this group desire an open-market employment [4], but for many this is never fulfilled. In a recent review on supported employment, approximately only one half of the study participants in the European context attained competitive employment [5]. Research has shown, however, that other forms of occupations, for example work practice, sheltered workshops and activities in day centers, also generate experiences of being part of a team, producing important products or services, contributing to others and developing one’s competencies [2, 6, 7].

Day centers and other community support programs thus play an important role for those who cannot manage open-market employment or who never get that opportunity. Many day centers and similar services have a goal of attendees developing self-confidence and skills for having an open-market employment or other more demanding everyday activities [8]. Research has indicated that there are generally two types of directions in day centers. One is meeting place-oriented where social and leisure activities dominate. The other is work-oriented where productive activities are central [9]. Although the characteristics of day centers may vary between cultures and societies there appear to be great similarities between those described in research from Canada [10], the United States [11], Europe [9, 12, 13] and Australia [14]. Important pathways through which day centers can promote productive activity include a clear focus on work-like activities within the services, user empowerment, opportunities for learning and a supporting social network [7].

There is an increasing focus on the rehabilitating potentials found in day centers. Tjörnstrand and colleagues [15] found that the activities could be graded so that the level of demands needed to perform the activity could be suited to the attendee’s needs and capacities. Swedish guidelines state explicitly that day centers should strengthen the attendees’ work capacity [16] and similar goals are found in international research where the users themselves express their needs and goals [3, 10, 17]. In order to follow whether efforts aimed at increasing attendees’ engagement in day center activities are successful, a screening tool that could be used by staff or researchers for evaluation purposes would be useful.

There are a few existing instruments that may be used to evaluate day center attendees’ engagement in activities. Profiles of Occupational Engagement in Severe mental illness – Productive occupations (POES-P) [18] was developed to evaluate day center attendees’ engagement in day center activities. It contains two parts, where the first is a time-use diary completed by the person with mental illness. The diary covers the hours spent in productive activities, such as those spent at a day center. In the second part the person with mental illness rates his or her level of engagement related to the diary content, using eight items. The attendee’s perspective on his or her engagement in activities is important for evaluations, which was the rationale for developing POES-P. This instrument was developed from the previous Profiles of Occupational Engagement in Severe mental illness (POES) [19, 20], which in contrast to POES-P covers everyday life activities over a period of 24 h and is completed by a professional. The POES-P rating can give the service providers better insight into the attendees’ performance and perceptions of their own engagement. It may also help the staff to address the attendees’ strengths and weaknesses from the attendees’ point of view and what their activity needs are. The POES-P can thus be viewed as a valuable tool in a day center setting to inform the future planning of productive activities, but also for monitoring any changes in the attendee’s self-reported occupational engagement. However, the staff who plan and organize the day center activities are in such a position as to be able to carry out appraisals of the attendees’ observed performance and progression [21]. Previous research on the ratings by mental health patients and staff, within areas such as quality of life [22] and the perceptions of the psychosocial ward climate [23], has shown that these two perspectives do not always concur and tend to reflect two different phenomena. Neither of the perspectives, however, appears to be superior to the other in terms of predicting care consumption [22]. The staff’s view of attendees’ engagement in day center activities can thus be said to highlight one side of the coin. Their perspective forms an important complement to the attendees’ ratings and needs to be included when organizing and planning for the day center services. This led us to develop a tool for capturing the staff perspective on the attendee’s engagement in activities, the General Occupational Engagement in people with Severe mental illness (GOES) scale. The GOES can assist the staff in systematizing their knowledge of the attendees’ capacities and, in that, using their professional skills in a goal-directed way. The POES-P reflects how the person with mental illness views his or her engagement in productive activities at the day center. By also using an instrument that reflects the staff perspective, such as GOES, possible discrepancies between the perspectives of attendees and the staff on a group level can be identified. These can serve as a basis for how to design the services in the best possible way. Although the attendees’ perceptions must be carefully considered in this process, the professionals’ perspective will add to the decision-basis. On the individual level, the staff perspective may serve a similar purpose and make the attendee reflect on his/her situation and progress with respect to engagement in the day center occupations. The aim of this study was thus to investigate the psychometric properties of GOES in terms of factor structure, internal consistency, item-total correlations, convergent and discriminant validity, and test-retest stability.

## Methods

### Study context and participants

The study, which was based on secondary analysis of data from two projects, was performed in the context of day centers designed for people with psychiatric disabilities. A psychiatric disability implies a lasting (>2 years) condition that seriously hinders a person in managing everyday life due to severe mental illness [24], but is not limited to certain diagnoses. The first data set (Sample 1) comprised seven day centers in the south of Sweden which were involved in a descriptive and comparative project [25]. Ninety-three persons visiting day centers were compared with a clinically equivalent group who did not have any structured daily activity. Data from all 93 persons who attended the day centers were used in the secondary analysis to assess all investigated psychometric properties of the GOES except test-retest stability. The second data set (Sample 2) was from a project evaluating a free-choice reform in the day center context [26]. Six day centers in a larger city in mid Sweden were included. Just as in Sample 1, the study participants completed instruments addressing various aspects of everyday activity and well-being. A baseline with two measurement points was established for a sub-sample of 41 participants (of a total of 123) before the reform was launched. These baseline data from Sample 2 were collected with a two-month interval. The original aim was to investigate baseline stability, before launch of the reform, regarding empowerment, engagement in activity, well-being and the like [26]. The GOES data from Sample 2 were also used in the current study to investigate the test-retest stability of the GOES. Sample 2 was not combined with Sample 1 because all variables needed to meet the study were not available for both samples. Sample 1 was thus used to address all analyses but test-retest stability of the GOES, whereas Sample 2 was used only for that purpose.

#### Recruitment procedure

Prior to recruitment of the participants, the projects were approved by the Regional Ethical Review Board in Lund (Reg. No. 303/2006 and Reg. No. 2009/625). The recruitment of participants followed the same procedure in both projects. The manager of each day center was first asked for participation at the center level. All but one of the day centers invited to Sample 1 and all invited to Sample 2 accepted. Information meetings were then held at each center to invite participants. In addition to the oral presentation of the project, written information was distributed. The information highlighted the study aim, what participation would entail, that participation was voluntary, that the participant could withdraw an approval at any time, and that data would be treated anonymously. All who agreed to participate gave their written informed consent. In both samples the participation rate was estimated at about 50% of those who attended the information meetings, which is in line with studies of comparable target groups [27, 28]. The exact proportion was not possible to calculate since people came and went during the meeting. Reasons for not choosing to participate were, for example, lack of motivation, tiredness and fear of having one’s responses registered. The data were collected between 2006 and 2010.

To help characterize the samples, self-reported diagnosis was asked for, which was subsequently coded into ICD-10 diagnoses by a specialized psychiatrist. Participant characteristics are shown in Table 1. Sample 2 was older (*p* = 0.027) and spent more hours in the day center (*p* = 0.018) but the samples did not differ in any other respects.

**Table 1:** Characteristics of the study participants

Characteristics	Sample 1*N* = 93	Sample 2^a^ *N* = 41
Age, mean (SD)	46 (10)	50 (10)
Gender: males; %	59	58
Civil status; married; %	19	17
Housing situation; %		
Independent living	64	73
Supported housing or housing support	36	21
Educational level; %		
Completed compulsory school or lower	47	33
Completed 6th form college or higher	53	67
Self-reported diagnoses %		
Schizophrenia and other psychosis	45	29
Mood disorders	21	29
Anxiety, phobia and stress disorders	20	18
Other diagnoses (mainly a personality or neuropsychiatric disorder)	15	24
Hours per week at the day center; mean (min-max)	13 (2–40)	17 (2–35)

The total number of participants varies somewhat between the variables due to missing data

^a^Sample 2 was used for test-retest stability only, whereas Sample 1 was used for all other analyses

### Data collection

Construct validity is the degree to which an instrument measures what it purports to measure [29]. The construct reflects the factors that underlie the construct [29], and one way of addressing these is to investigate the instrument’s factor structure. Convergent and discriminant validity are two other approaches for assessing construct validity. Convergent validity concerns the agreement between two constructs that, according to how they are defined and framed, are likely to be more-highly related to each other, while discriminant validity is evidenced by a lack of relationships between constructs that are assumed to be dissimilar or only weakly associated [30]. Both convergent and discriminant validity are typically assessed in terms of associations with other instruments [30]. Regarding discriminant validity, in addition to the phenomenon per se, there are additional factors that may maximize the difference, such as who is the rater. A self-report would be expected to differ from an observer’s rating. Similarly, regarding convergent validity, two self-reports on differing phenomena are likely to show some similarity because of the shared rater perspective. Different assessor perspectives thus tend to reflect different constructs [29–31].

#### The new tool

The POES-P [18] served as inspiration for the GOES. The same aspects of occupational engagement were addressed, but the items were slightly reworded, e.g. “I” was substituted with “The attendee” to reflect the staff perspective. This was performed jointly by the two authors. The wordings of the GOES items are; 1) The work tasks are adjusted to what the attendee can manage; 2) The attendee has a good balance between activity and rest/break; 3) The attendee can manage the work tasks on his/her own; 4) The attendee gets the support (s)he needs to manage the activity; 5) The attendee can manage to be with others in the day center; 6) The attendee takes the initiative for the activities (s)he performs; 7) The attendee has good routines in the activities (s)he performs; 8) The attendee appears to think what (s)he does is meaningful. No subscales are assumed, which is in accordance with both the POES [19, 32] and the POES-P [18]. No time-use diary is, however, used in the GOES. Instead, the staff member is instructed to think of what the attendee normally does and the activities (s)he performs at the day center before making the rating. This reflects one of the differences in relation to the POES-P, where the attendee rates his or her engagement in relation to the activities just performed. The other concerns the aforementioned difference in rater perspective. There are five response alternatives; 1 = not at all; 2 = seldom; 3 = sometimes: 4 = quite often; 5 = often. Based on the research assistants’ notes, it takes 5–10 min to complete the GOES and according to reactions from personnel, most staff members found it easy to understand and complete.

Five trained and skilled research assistants delivered the GOES to the staff, who got instructions regarding how to use the instrument and immediately completed the rating in a calm environment in the day center. The staff were not regarded as study participants in this study, but as a category of assessors, providing the staff perspective regarding the attendee’s engagement in day center activities. The staff member with most knowledge of an attendee’s situation at the day center performed the GOES rating.

GOES was the only instrument that was used with both samples in this study. All data generated from the instruments described below were from Sample 1. Regarding the less tested Motivation for day center participation (see below), Sample 2 was however used to assess its test-retest stability. The research assistants, who all had experience from working with the target group, performed the data collection with the attendees during personal meetings in a secluded room in the day center. For instruments based on interviews, the research assistants registered the ratings. Regarding self-reports, the research assistant supported the attendee in completing them, if needed, carefully trying to avoid influencing his or her responses. All instruments were administered in Swedish.

#### Instruments for assessing convergent validity

To the best of our knowledge, the POES-P [18] is the only instrument that targets engagement in day center activities and none seems to exist that employs the staff perspective. We thus had to rely on comparisons with related but not quite equivalent constructs regarding the assessment of convergent validity.

##### Satisfaction with Daily Occupations (SDO)

The nine-item SDO [33] was used. It is an interview-based instrument that assesses satisfaction with, and participation in, everyday activities in terms of work, leisure, domestic tasks, and self-care activities. Each of the nine items has two parts, the first addressing whether the person with mental illness presently (the past two months) participates in the activity addressed in the item. The response format is yes (=1) or no (=0) and all agreeing answers are summarized into an activity level score. This was considered a relevant variable to address convergent validity because of its association with occupational engagement according to previous research [19]. The person with mental illness also rates his or her satisfaction with presently participating, or not participating, in each of the indicated activities. These ratings form a satisfaction score, which has shown satisfactory internal consistency, construct validity and test-retest stability [34, 35]. This score was not seen as a relevant variable in relation to convergent validity, however, since satisfaction is a deeply personal experience and the GOES represents an outsider’s perspective. The present study thus only used the activity level score. Internal consistency is not a relevant evaluation in relation to the activity score since there is no concept of an underlying phenomenon, just a calculation of the number of occupations the person with mental illness is currently involved in. However, a correlation of 0.75 between the two measurement points for Sample 2 indicates good test-retest stability. The percentage of agreement was high at >95% for four items, 73–85% for four items and lower at 54% for one item (leisure on one’s own). A similar calculation was not possible for Sample 1, which participated on only one occasion. Further psychometric properties regarding the SDO activity scale are not known.

##### Motivation for day center participation

Assuming that motivation and engagement are related constructs, which has support in the self-determination theory [36], we also used items targeting motivation for participating in day center activities [37] to investigate convergent validity. From a set of four items, devised to investigate separate facets of motivation in the day center context and not forming a scale [26], we chose two items with clear reference to engagement in the day center activities, reflecting the proposed construct behind GOES. One item is worded “I set clear goals for the day center activities” and the other says “I would prefer to have a job to go to”. The first of these has obvious connections with engagement in day center activities, and the second was chosen because wanting a job may indicate a high level of activity engagement, particularly from a staff perspective. The staff in mental health community services tend to emphasize the importance of attendees engaging in work and other activities outside of the day center, even more than the attendees themselves [38, 39]. The response format for these items is a 100 mm line where the person with mental illness marks his or her rating, a so-called VAS (visual analogue scale). The items have been assessed as having face validity and content validity by a panel of mental health care users and has shown discriminant validity in terms of non-significant relationships with, for example, self-rated health and quality of life [27]. Test-retest stability was indicated in the current study by the fact that, based on Sample 2, no statistically significant differences were identified between the two measurement points regarding preferring a job (*p* = 0.437) or setting clear goals (*p* = 0.438). The corresponding correlations between the two measurement points were *r* = 0.53 (*p* = 0.001) and *r* = 0.37 (*p* = 0.017).

##### Worker Role Self-assessment (WRS)

The WRS [40] was also used. The worker role concerns energy and personal interests invested in activity, which makes the worker role construct similar to engagement in activities [41]. The WRS is a self-rating scale that targets both the person’s prospects of a future worker role and assets and routines for accomplishing such a role [42] and was therefore considered a pertinent variable for testing convergent validity. It is a self-report survey with 14 items addressing different aspects of the worker role, such as awareness of own abilities, taking responsibility for work tasks, wanting a future worker role, and ability to develop routines. The response scale has four alternatives from fully disagree (=1) to fully agree (=5). The WRS has shown acceptable internal consistency (alpha = 0.65–0.83), good content validity, and satisfactory test-retest reliability (item agreement varying from fair to very good according to kappa statistics) [40]. Cronbach’s alpha based on current Sample 1 was 0.85.

##### Hours spent in the day center

Finally, background data on hours per week spent in the day center was used to assess convergent validity, assuming that a longer presence in the day center might be indicative of a greater level of engagement in the activities there from a staff perspective.

#### Instruments for assessing discriminant validity

It was reasoned that variables pertaining to level of functioning and severity of psychiatric symptoms assessed by a researcher, as well as the attendee’s self-rated health, were phenomena that should be seen as dissimilar to engagement in activities. Furthermore, empirical research has shown that psychiatric symptoms and level of functioning are unrelated or weakly associated with various aspects of activity [43]. We also assumed that a measure of activity that deviates in both targeted activity and rater perspective could indicate discriminant validity.

##### Profiles of Occupational Engagement in Severe mental illness – Productive occupations (POES-P)

POES-P was used to assess discriminate validity because it deviates from the GOES in two ways. In contrast to the GOES it is based on the attendee’s report, and the rater perspective thus differs. Additionally, the POES-P reflects activity engagement during the most recent visit to the day center, which also means that the occupations are specified, whereas the GOES rating concerns the attendee’s engagement in the day center activities in general. In POES-P, the aforementioned time-use diary of the most recent visit to the day center is rated in terms of eight items focusing on work task adjustment, activity balance, ability to work independently, receiving support, ability to be with others, taking initiative, having good routines and perceiving meaningfulness. The response scale ranges from not at all (=1) to often (=5) and the POES-P has shown good internal consistency (alpha = 0. 85) and satisfactory construct validity in terms of convergent and discriminant validity [18]. Cronbach’s alpha based on the current Sample 1 was 0.93.

##### Global Assessment of Functioning scale (GAF)

Another instrument chosen to assess discriminant validity was the GAF scale [44, 45]. A professional (in this study one of the five research assistants) used a scale from 0–100 to rate the level of functioning of the person with mental illness. Psychometric research indicates the instrument has good inter-rater reliability and has shown concurrent validity in relation to psychiatric symptoms and social behavior [46]. As preparation for the data collection in Sample 1, one of the research assistants, who was also a clinician well-experienced in GAF ratings, trained the other four research assistants using videos. The training continued until acceptable inter-rater agreement (deviation of <10%) between the well-experienced research assistant and each of the other four was reached.

##### Brief Psychiatric Rating Scale (BPRS)

Severity of psychiatric symptoms was assessed by the 18-item BPRS [47]. An interview guide as proposed by Crippa and associates [48] was used. The items address general, depressive, and positive and negative psychotic symptoms. They are rated on a seven-point scale from no symptoms (=1) to severe symptoms (=7). Research indicates that the BPRS has inter-rater reliability [48] and discriminates between people with and without schizophrenia spectrum disorders [49]. A test of inter-rater reliability among the five research assistants trained for the data collection for Sample 1 showed an ICC of 0.85.

##### Self-rated health

The instrument addressing *self-rated health* was the first item from the Medical Outcome Scales SF-36 [50]. It correlates highly with the scale as a whole [51] and has been argued to be a reliable evaluation of self-rated health [52]. It uses a score from 1 to 5 where a low score indicates very good health. A correlation of −0.70 with general well-being in Sample 1 suggests good construct validity.

### Data analysis

The following hypotheses were postulated:

- The GOES would form a one-factor scale with internal consistency exceeding 0.70 (Sample1).
- The GOES would exhibit moderate or high positive correlations to the instruments chosen to investigate convergent validity (Sample 1). The constructs behind these instruments have been shown to be related with activity engagement in previous research [18, 20, 42].
- The GOES would exhibit low correlations with the instruments selected to address discriminant validity (Sample 1), as indicated by previous research on relationships between activity engagement and these constructs [18].
- The GOES would exhibit test-retest stability as indicated by moderate to high positive correlations between measurement points and an absence of large changes (Sample 2).

Explorative factor analysis was applied. In line with recommendations [53], an eigenvalue of 1 and Scree plot were used to identify the number of factor(s) and an item loading of 0.40 or more to a component was seen as contributing to saturation of that component [54]. The Kaiser-Meyer-Olkin (KMO) measure of sampling adequacy and Bartlett’s test of sphericity were used to test if data were suitable for factor analysis. KMO should be >0.6 and Bartlett’s test of sphericity should be statistically significant [53]. The Cronbach’s alpha test and corrected item-total correlations (CITC) [55] were performed to investigate internal consistency. Levels for satisfactory values are 0.80 for internal consistency and 0.30 for CITC [29]. Pearson correlations were calculated to test for convergent and discriminant validity. The limits for moderate and strong correlations were set at >0.30 for a moderate correlation and >0.50 for a strong correlation [56]. The paired-samples *t*-test was employed to assess test-retest stability, but we also calculated effect sizes to get an estimate of strength of differences. The formula for Cohen’s *d,* (*M*_1_ – *M*_2_) ⁄*SD*_pooled_, was used. The mean from measurement 2 was thus subtracted from the mean of measurement 1 and the rest divided by the pooled standard deviation [55]. Effect sizes ≤0.2 are considered small, 0.3–0.7 as medium and >0.8 as large [56]. The software used was SPSS, version 22, and the *p*-value was set at 0.05.

The number of participants needed for psychometric testing is contingent on the type of analyses used. Among the analyses employed in the current study, factor analysis is the one that requires the largest sample. According to methodological literature, the number of participants should be 5–10 times the number of items [57], thus ≥80 for the current study.

## Results

### Factor solution and internal consistency

**Fig. 1:**
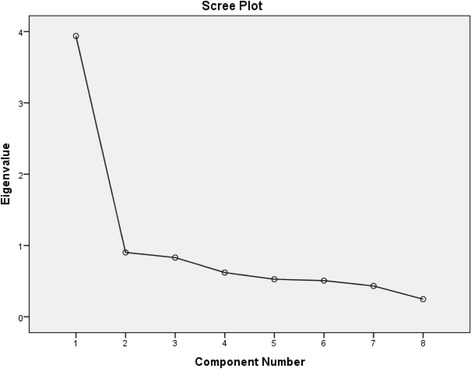
Scree plot from the exploratory factor analysis

A one-factor solution was found for the eight GOES items, confirming our first hypothesis. A factor with an eigenvalue of 3.9 explained 49.2% of the variation. The Scree plot clearly indicated one factor (see Fig. 1), with all remaining factors having an eigenvalue below 1. KMO was 0.82 and Bartlett’s test of sphericity was statistically significant at *p* < 0.001. All items showed a loading above 0.40 to the identified factor, as shown in Table 2. When testing internal consistency, a Cronbach’s alpha coefficient of 0.85 was obtained and all CITC ranged between 0.56 and 0.65, which was also in line with our first hypothesis.

**Table 2:** The component matrix from the factor analysis and the participants’ mean (SD) ratings on the respective items

	Component 1, factor loadings	Mean rating (SD)
GOES 1 – work tasks adjusted to the attendee	0.673	4.2 (1)
GOES 2 – good balance between activity and rest/break	0.697	4.1 (1)
GOES 3 – attendee can manage the work tasks	0.700	4 (1.1)
GOES 4 – attendee gets the support needed	0.672	4.2 (0.9)
GOES 5 – attendee can manage to be with others	0.677	4 (1)
GOES 6 – attendee takes initiative for activities	0.761	3.9 (1)
GOES 7 – attendee has good routines	0.722	4 (1)
GOES 8 – what attendee does seems meaningful	0.705	4.2 (1)

*GOES* General Occupational Engagement in people with Severe mental illness

### Convergent and discriminant validity

Correlations with the selected variables are shown in Table 3, where it can be seen that the GOES showed the highest correlation with Clear goals for participating in the day center activities. Associations indicating varying degrees of convergent validity were also found with Activity level, Prefer open-market employment, Worker role, and Hours/week spent at the day center, although somewhat lower than hypothesized. As hypothesized and indicating discriminant validity, GOES was not associated with the POES-P, Self-rated health, GAF or BPRS. Table 4 displays correlations between the variables used to assess convergent and discriminant validity. As presented there, only one correlation, that between GAF and BPRS, was in the realm of a strong correlation, thus indicating that none of the variables was redundant, except for possibly one of these two.

**Table 3:** Correlations between GOES and the variables chosen to evaluate convergent and discriminant validity

	Coefficient	*P*-value
Indicators of convergent validity		
Activity level (based on the SDO)	*r* = 0.23	*p* = 0.034
Clear goals (motivation aspect)	*r* = 0.47	*p* < 0.001
Prefer work (motivation aspect)	*r* = 0.33	*p* = 0.003
Worker role (based on WRS)	*r* = 0.26	*p* = 0.019
Hours/ week (spent at the day center)	*r* = 0.27	*p* = 0.015
Indicators of discriminant validity		
POES-P (attendee perceptions of engagement)	*r* = 0.04	ns.
Self-rated health (single SF-36 item)	*r* = −0.06	ns.
GAF (psychosocial functioning)	*r* = −0.04	ns.
BPRS (psychiatric symptoms)	*r* = −0.16	ns.

*GOES* General Occupational Engagement in people with Severe mental illness, *SDO* Satisfaction with Daily Occupations, *WRS* Worker Role Self-assessment, *POES-P* Profiles of Occupational Engagement in Severe mental illness – Productive occupations, *GAF* Global Assessment of Functioning, *BPRS* Brief Psychiatric Rating Scale

**Table 4:** Correlations between the variables selected to assess convergent and discriminant validity

	Activity level	Clear goals	Prefer work	Worker role	Hours/ week	POES-P	Self-rated health	GAF
Clear goals	0.10							
Prefer work	0.12	0.40***						
Worker role	0.26*	0.34**	0.36**					
Hours/ week	0.28**	−0.001	−0.003	0.23*				
POES-P	0.13	0.19	−0.01	0.36**	0.42***			
Self-rated health	−0.09	−0.28**	−0.20	−0.32**	−0.15	−0.39***		
GAF	0.14	0.05	0.02	0.06	0.24*	0.22*	−0.11	
BPRS	−0.11	−0.13	−0.02	−0.15	−0.32**	−0.47***	0.21	−0.55***

* *p* < 0.05; ** *p* < 0.01; *** *p* < 0.001

*POES-P* Profiles of Occupational Engagement in Severe mental illness – Productive occupations, *GAF* Global Assessment of Functioning, *BPRS* Brief Psychiatric Rating Scale

In order to ascertain that the low correlation between the GOES and POES-P did not obscure stronger relationships between single items, each GOES item was correlated with the corresponding POES-P item. All associations were found to be low, but items 6 (targeting initiatives; *r* = 0.26, *p* = 0.022) and items 8 (targeting meaningfulness; *r* = 0.27, *p* = 0.015) in the respective scales showed inter-correlations marginally below the level for a moderate relationship. The other item-to-item correlations varied between *r* = −0.005 and 0.176.

### Test-retest stability

Based on Sample 2, no difference between the two measurement points was identified on scale level (*p* = 0.276). The effect size was 0.17. One item, number 4 targeting getting the support needed, showed a statistically significant difference at *p* = 0.04, revealing a decrease in the rating on the second occasion. The effect size was 0.34. The other item differences obtained effect sizes varying between 0 (item 1; *p* = 1) and 0.26 (item 2; *p* = 0.117). The correlation on scale level between the two measurement points was *r* = 0.32 (*p* = 0.043). In all, this suggests test-retest stability of the GOES and confirmed our final hypothesis.

## Discussion

The factor analysis indicated one factor, which was in line with the intended design of the scale and the hypothesis. This supported that the GOES taps a uniform construct. The alpha value obtained of above 0.80 and all CITC well above 0.30 further indicate a homogeneous scale. In fact, the high CITC values, which were all above 0.50, indicates possible redundancy [29] and it could be considered whether some items could be deleted.

The findings gave a clear picture of discriminant validity, i.e. what the GOES is *not* about, since all correlations assumed to show no or low associations with the selected constructs met the hypotheses. Staff perceptions of engagement in activities (GOES) were obviously not related with level of functioning (GAF), symptomatology (BPRS) or health, as addressed from different rater perspectives (the researcher and the person with mental illness). This is in line with other research showing that instruments reflecting activity are only weakly correlated with measures of psychopathology and functioning [58]. Nor was the GOES related with attendees’ ratings of engagement in recent activities (POES-P). That different rater perspectives, such as staff and service users, differ so much that they must be regarded as reflecting separate phenomena has been argued in both the methodological literature [29] and in theoretical and empirical studies within the mental health care field [30, 31]. In the case of occupational engagement, an additional rater perspective, such as the staff perspective reflected in GOES, is a strength in itself. When used cautiously it may enrich the discussion with the user and thus contribute to the creation of meaningful activities for the latter.

It is the convergent validity that indicates what an instrument really *is* about. The correlations with the selected variables were in line with the hypothesis, although not all were in the range of moderate or high according to the limits proposed by Wampold [56]. The highest correlation concerned the attendee’s rating of motivation in terms of setting clear goals for what to do in the day center. Although the rater perspectives differed, the size of the correlation was on the border to a strong correlation. This pinpoints that the staff perspective of the attendee’s engagement in activities mainly concerns working towards clear goals. Additional facets concerned the attendee’s orientation towards the worker role and employment (based on WRS and motivation in terms of preferring work), his or her level of activity (based on SDO), and the number of hours spent at the day center. The sizes of the correlations were in line with other instrument studies using related but not quite equivalent variables to investigate convergent validity of activity assessments [18, 59].

The fact that the GOES was compared with variables based on attendee ratings to address convergent validity makes the medium-sized correlations comprehensible. Given the difference in rater perspective, stronger associations than that would not be expected. Interestingly, when both the perspective (staff/attendee) and the time frame (in general/ activities during the previous visit) differed, the common denominator of “activity engagement” was not enough to produce anything but a weak relationship, as in the case of the correlation between the GOES and the POES-P.

The GOES showed fair stability between two measurement occasions that served as a baseline for the evaluation of an imminent free-choice reform. A very small effect size for change between the measurement points was seen on the scale level. Item 4, addressing the support offered to the attendees, showed a medium-sized change, with the staff scoring lower on the second occasion. It cannot be excluded that this may reflect staff worries about the coming reform, demonstrated in a qualitative study following the reform [60]. The other seven items showed stability, but a smaller gap than the current two-month interval would have been more ideal for a test-retest stability assessment.

Further tests of construct validity of the GOES are needed, such as criterion and predictive validity. It would be interesting to know whether GOES could, for example, discern people with employment from those without and predict future employment. Additional tests of convergent validity are needed as well, but the findings from this first test taken together indicate encouraging properties regarding construct validity and scale homogeneity.

### Methodological considerations

It could be argued that the sample of this study was small for a factor analysis, but according to the rule of 5–10 participants per item [57] it was sufficient. The values for KMO and Bartlett’s test of sphericity were both well within stipulated intervals [53], which further supports the trustworthiness of the factor analysis. A more obvious drawback is the lack of an instrument possessing a gold standard with respect to staff perceptions of engagement in day center activities. The GOES may be seen as a start of instrument development in that area, however, and the variables included in this study were the most appropriate choice of those that are presently available and were known to the research team. As already mentioned, the time interval between the two measurement points was not optimal, and the stability indicated should be seen as a preliminary finding. Finally, it must be seen as a limitation that the current study did not address inter-rater agreement. This is an urgent task in further development of the GOES.

## Conclusions

Meaningful everyday activities, either productive or more social and leisure-oriented, are increasingly in focus in psychiatric rehabilitation. An instrument addressing staff perceptions of engagement in day center activities would be an important contribution to the possibilities for evaluating and further developing such practices. The GOES may function as a tool in such endeavors and serve as a supplement to attendees’ self-reported occupational engagement. Although its construct validity and test-retest stability needs further exploration these first findings appear to be promising and the GOES is ready for use in rehabilitation services and research where productive and other types of activities are of interest.

## Abbreviations

BPRS: Brief Psychiatric Rating Scale
CITC: Corrected Item-Total Correlations
GAF: Global Assessment of Functioning
GOES: General Occupational Engagement in people with Severe mental illness
POES: Profiles of Occupational Engagement in people with Severe mental illness
POES-P: Profiles of Occupational Engagement in people with Severe mental illness – Productive occupations
SDO: Satisfaction with Daily Occupations
VAS: Visual Analogue Scale
WRS: Worker Role Self-assessment
